# Quality of Life in Yoga Experienced and Yoga Naïve Asian Indian Adults with Obesity

**DOI:** 10.1155/2019/9895074

**Published:** 2019-04-30

**Authors:** Shirley Telles, Sachin Kumar Sharma, Alok Singh, Niranjan Kala, Vikas Upadhyay, Jaideep Arya, Acharya Balkrishna

**Affiliations:** ^1^Patanjali Research Foundation, Haridwar, India; ^2^Patanjali Yog Samiti, Haridwar, India

## Abstract

**Background:**

Obesity adversely affects quality of life which then acts as a barrier to weight loss and weight loss maintenance. Hence, those interventions which positively influence the quality of life along with weight reduction are considered useful for sustained weight loss in persons with obesity. An earlier study showed better quality of life in obese adults who had experience of yoga compared to yoga naïve obese adults. However, the main limitation of the study was the small sample size (*n*=20 in each group).

**Objective:**

The present study aimed to determine whether with larger sample sizes the quality of life would differ in yoga experienced compared to yoga naïve adults with obesity.

**Methods:**

There were 596 Asian Indian obese adults (age range 20 to 59 years; group mean age ± SD; 43.9 ± 9.9 years): of whom (i) 298 were yoga experienced (154 females; group mean age ± SD; 44.0 ± 9.8 years) with a minimum of 1 month of experience in yoga practice and (ii) 298 were yoga naïve (154 females; group mean age ± SD; 43.8 ± 10.0 years). All the participants were assessed for quality of life using the Moorehead–Ardelt quality of life questionnaire II. Data were drawn from a larger nationwide trial which assessed the effects of yoga compared to nutritional advice on obesity over a one-year follow-up period (CTRI/2018/05/014077).

**Results:**

There were higher participant-reported outcomes for four out of six aspects of quality of life in the yoga experienced compared to the yoga naïve (*p* < 0.008, based on *t* values of the least squares linear regression analyses, Bonferroni adjusted, and adjusted for age, gender, and BMI as covariates). These were enjoyment in physical activities, ability to work, self-esteem, and social satisfaction.

**Conclusion:**

Obese adults with yoga experience appear to have better quality of life in specific aspects, compared to yoga naïve persons with a comparable degree of obesity.

## 1. Introduction

As of June 2017, the Asia Pacific region had the largest absolute number of overweight and obese people equivalent to one billion [1]. In these regions, two out of every five adults are either overweight or obese [1]. Various aspects of quality of life are impaired in persons with obesity; these include low self-esteem, impaired psychosocial functions, disability, reduced physical activity, and sexual dysfunction [2–7]. It has been reported that the quality of life improves after intentional weight loss in persons with obesity [8, 9].

However, this is not always the case. For example, weight loss through severe calorie restriction, use of laxatives, diuretics, and excessive exercise is associated with decreased health-related quality of life in persons with obesity [10–12]. Hence, those interventions which could positively influence health-related quality of life and measures of obesity (e.g., a reduction in BMI and waist circumference) are considered clinically useful for persons with obesity [13]. Examples of clinically useful interventions include increased physical activity and a healthy diet [13]. However, it is known that obese persons experience several challenges in initiating and adhering to increased physical activity [14, 15]. Therefore, pragmatic interventions which increase the level of physical activity and are easy to follow have been recommended for sustained weight loss in obese persons [13].

Yoga is one such intervention with studies reporting long-term adherence and benefits in various health conditions including obesity [16, 17]. Effects of yoga on the quality of life were seen in a single-arm interventional study on 279 overweight and obese Asian Indian persons of both sexes aged between 20 and 60 years who showed a significant improvement in physical, psychological, and environmental domains of the quality of life based on the World Health Organization Quality of Life Instruments (WHOQOL-BREF) questionnaire, after 10 days of a yoga-based lifestyle intervention [18].

Apart from this, in a cross-sectional study, twenty obese Asian Indian obese adults of both sexes who had experience of yoga were compared with an equal number of yoga naïve obese persons of both sexes for six domains of quality of life in the Moorehead–Ardelt quality of life questionnaire II (i.e., enjoying physical activities, ability to work, self-esteem, social satisfaction, sexual pleasure, and approach towards food) and overall quality of life [19]. The yoga group showed significant improvements in three of the six domains (i.e., enjoying physical activities, ability to work, and self-esteem) and overall quality of life compared to the yoga naïve group. The main limitation of the study [19] was the small sample size (*n*=20; in each group).

Hence, the present cross-sectional study was planned primarily to compare the overall quality of life using the Moorehead–Ardelt quality of life questionnaire II in a larger sample of Asian Indian obese persons (*n*=596), of both sexes, of whom 298 had experience in practicing yoga, whereas 298 were yoga naïve. The secondary aim of the study was to compare the six different subdomains (i.e., general self-esteem, enjoyment in physical activities, satisfactory social contacts, satisfaction concerning work, sexual pleasure, and focus on eating behavior) of the Moorehead–Ardelt quality of life questionnaire II in the same sample of yoga experienced and yoga naïve obese adults (*n*=596).

## 2. Materials and Methods

### 2.1. Study Design

The present data were taken as part of a larger trial conducted across India to compare the effects of yoga with nutritional advice over a one-year follow-up period (CTRI/2018/05/014077). At the time of recruitment in 55 centers across India, participants were asked whether they had prior experience of yoga practice or not, as only yoga naïve persons who did not plan to adopt any other physical activity regimen were included in the larger trial. The present data were collected during the recruitment of participants for the larger trial, for which data analysis is still on-going.

The present study was a secondary analysis of data collected for screening eligibility criteria of an interventional trial designed to assess the effects of yoga and nutritional advice on obese adults. The screening for the interventional trial was done in two phases.

*Phase 1*: the participants were assessed for anthropometry (i.e., weight and height using standard methods) to determine their body mass index (BMI). Those with BMI ≥25 kg/m^2^ were considered for the second phase.

*Phase 2*: In this phase, the participants were screened for the following: (i) any metabolic abnormality (e.g., hypothyroidism), (ii) obesity secondary to hormonal imbalance, secondary to medications such as steroids, or secondary to any other medical conditions, and (iii) any psychiatric illness (e.g., depression). At this stage, all the participants gave their signed informed consent to be included in the trial, and if they did not meet the criteria, their consent included using their data for research. All of them provided their sociodemographic details and filled in the Moorehead–Ardelt Quality of life questionnaire II at this stage. The sociodemographic form included their name, age, gender, occupation, years of education, and other details. Participants were asked a single question about yoga practice, viz., “Do you practice yoga? If “yes,” please mention the duration in months” (where yoga meant yoga postures (*asanas*), yoga breathing (*pranayamas*), and/or yoga meditation (*dhyana*)). Based on this response, the participants were categorized as yoga experienced or yoga naïve. Equal numbers of yoga experienced and yoga naïve participants were selected for comparison from the larger data set. The person who selected the participants had no access to the quality of life scores data, and hence, the selector was blinded to these scores but matched participants of both groups for their age, gender, and body mass index (BMI).

### 2.2. Participants

There were five hundred and ninety-six obese adults (age range between 20 and 59 years; group mean age ± SD; 43.9 ± 9.9 years): of whom 298 were yoga experienced (154 females; with group mean age ± SD; 44.0 ± 9.8 years) with a minimum of 1 month of experience in yoga practice, an mean of 3.2 ± 4.7 years, and a range of experience from 1 to 480 months. The remaining 298 obese adults (154 females; group mean age ± SD; 43.8 ± 10.0 years) did not have any prior experience in yoga practice and were described as yoga naïve. In the present study, a priori calculation of the sample size was not done. However, the *post hoc* analyses showed that, for the present study, with the sample size of 298 in each group and Cohen's *d* of 0.40 calculated from the mean and SD of the overall quality of life score, the power was 0.998201. Inclusion criteria were as follows: (i) BMI ≥ 25 kg/m^2^ [20] and (ii) age range between 20 and 59 years. Exclusion criteria were as follows: (i) any metabolic abnormalities (e.g., hypothyroidism), (ii) obesity secondary to hormonal imbalance, medication such as steroids or secondary to any other medical condition, (iii) any psychiatric illness (e.g., depression), and (iv) incompletely filled in Moorehead–Ardelt quality of life questionnaires II. None of the participants had to be excluded for these reasons. The study had the approval of the institution's ethical committee (Approval number YRD-017/022).

### 2.3. Assessment

#### 2.3.1. Quality of Life

The quality of life was assessed using the Moorehead–Ardelt quality of life questionnaire II [21]. The questionnaire has been used to assess the quality of life in overweight and obese persons in India [16, 19], where the present study was carried out. In addition, the questionnaire is not culture sensitive [22] and has been used in different countries to assess the quality of life in obese persons [22, 23]. The questionnaire is designed to assess different aspects of quality of life such as general self-esteem, enjoyment in physical activities, satisfactory social contacts, satisfaction concerning work, sexual pleasure, and focus on eating behavior, on an equally weighted 10-point Likert scale with scores ranging from −0.5 to +0.5 [21]. The sum of these 6 scores provides an overall quality of life score. In the present study, English version of the questionnaire was used. However, for those who were not able to understand English, the questionnaire was translated in the local language, i.e., Hindi, by two language experts as follows: two independent bilingual experts translated the questionnaire from English to Hindi. Discrepancies between the two translators were discussed and resolved by the translators. After this, Hindi version was back translated to English by two independent translators to ensure the accuracy of the translation. Unclear wordings or any misunderstandings were again resolved by mutual discussion.

### 2.4. Statistical Methods

The analyses of the data were carried out using PASW (Version 18.0, SPSS Inc). Three types of analyses were performed which are mentioned below.

#### 2.4.1. Q-Q (Quantile-Quantile) Plots for Normal Distribution

The data (overall quality of life scores and six subdomains) were tested for normal distribution using Q-Q plots.

#### 2.4.2. Chi-Square Test

Ages and BMI values of the two groups were compared using the chi-square test.

#### 2.4.3. Regression Analysis

*(1) Groups, Quality of Life Scores, and Three Covariates*. The data of the two groups (i.e., yoga and yoga naïve groups) were compared using the least squares regression analysis adjusted for three covariates, i.e., age, gender, and BMI. These covariates were selected based on the outcomes of the earlier studies [24–26]. Separate linear regression models were used to compare the two groups for (i) overall quality of life and (ii) the six subdomains of the quality of life (i.e., enjoying physical activities, ability to work, self-esteem, social satisfaction, sexual pleasure, and focus on eating behavior). In each model, scores of either overall quality of life or one of the six subdomains of the quality of life acted as the dependent variable. Statistical significance (*α*) and confidence interval (CI) were Bonferroni adjusted and set at 0.008 and 99.2 percent, respectively, when analyzing the six subdomains of the Moorhead–Ardelt Quality of Life Questionnaire II.

*(2) Yoga Experience in Months, Overall Quality of Life Scores, and Three Covariates*. The association between duration of yoga experience in months with overall quality of life scores was evaluated using least squares regression analysis adjusted for three covariates, i.e., age, gender, and BMI.

## 3. Results

The details of baseline characteristics of both groups are mentioned in Table 1.

### 3.1. Q-Q (Quantile-Quantile) Plots for Normal Distribution

Visual inspection of the Q-Q plots showed that data were not normally distributed. However, the parametric test (least squares regression) was used to compare the data of the two groups as parametric tests are considered robust enough for a large sample size [27].

### 3.2. Chi-Square Test

At baseline, there were no statistically significant differences between the yoga (*n*=298) and yoga naïve (*n*=298) groups, for the following variables: (i) age (*χ*^2^ = 0.02, *p*=0.99) and (ii) BMI (*χ*^2^ = 1.54, *p*=0.21).

### 3.3. Regression Analysis

#### 3.3.1. Groups, Quality of Life Scores, and Three Covariates

**Figure 1:**
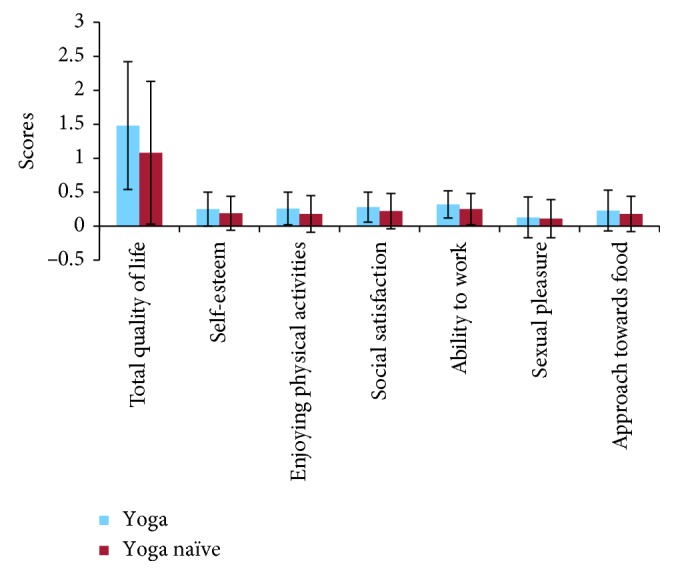
Graphical presentation of adjusted mean scores of quality of life of the yoga experienced and yoga naïve obese adults. Error bar showing the standard deviation of the quality of life scores.

Least squares regression analysis adjusted for three covariates (i.e., age, gender, and BMI) showed that there was a statistically significant difference between the groups for overall quality of life (*t* = 4.825, *p* < 0.001) (Figure 1). Also, the least squares regression analyses adjusted for the three covariates showed a statistically significant difference between the groups for (i) enjoying physical activities (*t* = 4.172, *p* < 0.001), (ii) ability to work (*t* = 4.465, *p* < 0.001), (iii) self-esteem (*t* = 2.976, *p*=0.003), and (iv) social satisfaction (*t* = 3.295, *p*=0.001). The details of the analyses are provided in Table 2. Also, the group mean values ± SD scores along with Cohen's *d* (an effect size used to indicate the standardized difference between two means) of quality of life for both groups are given in Table 3.

#### 3.3.2. Yoga Experience in Months, Overall Quality of Life Scores, and Three Covariates

Least squares regression analysis adjusted for three covariates (i.e., age, gender, and BMI) showed no statistically significant association (*F* = 4.965, df = 4,293, adjusted *R*^2^ = 0.05) of yoga experience in months with overall quality of life scores (*β* = −0.075, *p*=0.202).

## 4. Discussion

Higher participant-reported outcomes were found in the overall quality of life and in four subdomains of quality of life in persons with obesity who had experience in yoga compared to obese persons who were yoga naïve. These subdomains of quality of life were enjoyment in physical activities, ability to work, self-esteem, and social satisfaction.

The overall quality of life was significantly better in yoga experienced obese persons compared to the yoga naïve. The magnitude of difference between the two groups based on Cohen's *d* was 0.40, which is considered as average [28]. Given that four out of six subdomains of the Moorehead–Ardelt quality of life questionnaire II (i.e., enjoyment in physical activities, ability to work, self-esteem, and social satisfaction) were higher in yoga experienced obese persons, the overall score of quality of life which is the sum of all the scores could be expected to be higher in yoga experienced obese persons.

Previously, a ten-day longitudinal trial reported the effects of an integrated yoga module which included yoga practice and theory for overweight and obese persons [18]. This single-arm interventional trial reported better physical, psychological, and environmental dimensions of the WHOQOL-BREF questionnaire following yoga. There was a single group of 279 participants of both sexes who did not differ significantly. The improvement in the physical dimension of quality of life is comparable to the increased enjoyment of physical activities seen in yoga practitioners in the present trial.

This higher self-reported enjoyment of physical activities in the present study could be explained by the outcomes of previous studies on yoga for obesity. In a fifteen-day comparative controlled trial, yoga reduced the body mass index and waist circumference, while the ability to balance and the handgrip strength increased [29]. A randomized controlled trial assessing the effects of yoga on female adults with abdominal obesity reported a reduction in perceived stress levels along with other favourable changes in mental health-related outcomes such as the health-related quality of life, self-esteem, better body awareness, and trust in bodily sensations following twelve weeks of yoga [30]. The results of these studies indicate that yoga decreases physical and psychological efforts required to be physically active in persons with obesity.

Obesity adversely affects workplace productivity [31]. Persons with obesity were more likely to be absent from their workplace and less productive while at work due to health-related conditions associated with obesity [32, 33]. Participation in a lifestyle intervention which included increased physical activity was reported to enhance workplace productivity by improving physical and mental health in the obese [34]. With these health benefits, absenteeism due to sickness decreases and enhances the ability to work better [31].

In an earlier study mentioned above [18], higher levels of psychological well-being in the WHOQOL-BREF questionnaire can be considered to be partially based on higher levels of self-esteem in yoga experienced persons compared to those who are yoga naïve. Practicing gives specific emphasis to body awareness and responsiveness to self-objectification [35]. When body awareness and responsiveness to how the body is viewed increases, there is a greater sense of body satisfaction and lesser chances of self-objectification. These factors could have contributed to the better self-esteem in yoga experienced obese persons. Improved self-esteem could in turn influence interaction with other persons. Yoga practice creates more interpersonal interactions [36, 37]. These factors also increase mental well-being associated with yoga practice [36, 37] which could explain the higher levels of social satisfaction in the yoga experienced compared to the yoga naïve participants. These findings, i.e., better social satisfaction were not observed in the earlier study conducted on smaller numbers (*n*=20, each group) of yoga experienced and yoga naïve obese persons [19].

The significance of present findings is that if a person who is obese enjoys physical activities, they are likely to adhere to any physical activity program including yoga which would be definitely beneficial to maintain and possibly further weight loss.

A limitation of the present cross-sectional study is that factors other than yoga could have influenced the results. There was no association between the duration of yoga practice and overall quality of life scores. However, adequate details were not obtained about the frequency of yoga practice in terms of number of days in a week or the intensity of the yoga practice based on number of minutes of practice in a day. Also, while “yoga” included physical postures (*asanas*), regulated breathing (*pranayamas*), and yoga meditation (*dhyana*), the exact details about the school of yoga followed were not obtained. It would have been ideal to know these details and take them into account. Hence, the results suggest that practicing yoga possibly influences the quality of life in obese persons though the quantum of practice does not appear to influence the results.

## Figures and Tables

**Table 1:** Baseline characteristic profile of participants: yoga experienced and yoga naïve group.

Characteristics of participants	Yoga group	Yoga naïve group
Number (*n*)	298	298
*Age (years): mean* *±* *SD*	44.0 ± 9.8	43.8 ± 10.0
20 to 30 years, *n* (%)	35 (11.7)	34 (11.4)
31 to 50 years, *n* (%)	175 (58.7)	175 (58.7)
51 to 59 years, *n* (%)	88 (29.5)	89 (29.9)

*Age range (years)*	20–59	20–59
BMI (kg/m^2^): mean ± SD	32.1 ± 4.5	32.3 ± 4.5
25.0 to 32.4 kg/m^2^: *n* (%)	178 (59.7)	163 (54.7)
≥32.5 kg/m^2^: *n* (%)	120 (40.3)	135 (45.3)

*Gender*		
Male : female	144 : 154	144 : 154
Percentage values	48.3 : 51.7	48.3 : 51.7

*Years of education: n (%)*		
<10 years	50 (16.8)	78 (26.2)
10–12 years	43 (14.4)	41 (13.8)
>12 years	205 (68.8)	179 (60.0)

*Marital status: n (%)*		
Married	264 (88.6)	262 (87.9)
Unmarried	29 (9.7)	23 (7.7)
Widow/widower	3 (1)	8 (2.7)

*Occupation information: n (%)*		
Business	87 (29.2)	82 (27.5)
Agriculture	7 (2.3)	19 (6.4)
Household	81 (27.2)	71 (23.8)
Professionals	52 (17.4)	40 (13.4)
Secretarial/clerical/officers	43 (14.4)	47 (15.8)
Self-employed	14 (4.7)	21 (7)
Skilled labour	1 (0.3)	2 (0.7)
Not mentioned	13 (4.4)	16 (5.4)

*Socioeconomic information: n (%)*		
Low income	43 (14.4)	52 (17.4)
Pre-middle income	98 (32.9)	103 (34.6)
Middle income	91 (30.5)	83 (27.9)
High income	52 (17.4)	48 (16.1)
Not mentioned	14 (4.7)	12 (4.0)

*Dietary information: n (%)*		
Vegetarian	211 (70.8)	187 (62.8)
Nonvegetarian	87 (29.2)	111 (37.2)

*Consumption of addictive substances (alcohol and/or tobacco): n (%)*		
Yes	26 (8.7)	39 (13.1)
No	253 (84.9)	248 (83.2)
Not mentioned	19 (6.4)	11 (3.7)

**Table 2:** Details of the regression analyses adjusted for the three covariates (age, gender, and BMI) for overall quality of life and six subdomains.

Quality of life	*F*	df	Adjusted *R*^2^	Related to covariates		
				Covariates	*β*	*p* value
Total quality of life	8.547	1, 590	0.048	Age	0.024	0.557
				Gender	−0.029	0.479
				BMI	−0.116	0.005

Enjoying physical activities	5.253	1, 591	0.001	Age	0.007	0.869
				Gender	0.015	0.727
				BMI	−0.074	0.072

Ability to work	7.970	1, 591	0.045	Age	−0.075	0.066
				Gender	0.046	0.269
				BMI	−0.109	0.008

Self-esteem	4.953	1, 591	0.026	Age	0.070	0.090
				Gender	−0.020	0.641
				BMI	−0.104	0.012

Social satisfaction	3.634	1, 591	0.017	Age	0.052	0.211
				Gender	−0.026	0.532
				BMI	−0.039	0.350

Sexual pleasure	8.986	1, 591	0.051	Age	−0.810	0.418
				Gender	−5.435	<0.001
				BMI	−1.232	0.218

Approach towards food	4.237	1, 591	0.021	Age	0.095	0.021
				Gender	0.090	0.032
				BMI	−0.085	0.041

**Table 3:** Quality of life scores in yoga experienced and yoga naïve persons with obesity.

Group as a whole								
Overall quality of life and subdomains	Yoga (*n*=298)		Yoga naïve (*n*=298)		Cohen's *d*	Mean difference	*t* value	*p* value^##^
	Mean ± SD	95% CI^#^	Mean ± SD	95% CI^#^				
Total quality of life	1.5 ± 0.94	1.39, 1.61	1.1 ± 1.05^@^	0.98, 1.22	0.40	0.40	4.82	<0.001
Enjoying physical activities	0.27 ± 0.24	0.24, 0.3	0.18 ± 0.27^*∗∗*^	0.15, 0.21	0.35	0.09	4.17	<0.001
Ability to work	0.34 ± 0.2	0.32, 0.36	0.26 ± 0.23^*∗∗*^	0.23, 0.29	0.37	0.08	4.46	<0.001
Self-esteem	0.25 ± 0.25	0.22, 0.28	0.19 ± 0.25^*∗*^	0.16, 0.22	0.24	0.06	2.98	0.003
Social satisfaction	0.29 ± 0.22	0.27, 0.32	0.23 ± 0.26^*∗*^	0.2, 0.26	0.25	0.06	3.29	0.001
Sexual pleasure	0.15 ± 0.3	0.12, 0.18	0.13 ± 0.28	0.1, 0.16	0.07	0.02	0.87	0.384
Approach towards food	0.25 ± 0.3	0.22, 0.28	0.19 ± 0.26	0.16, 0.22	0.21	0.06	2.32	0.021

^@^ *p* < 0.001 at the two-tailed level, level of statistical significance between the groups was analysed using separate least squares regression. ^*∗*^*p* < 0.008 and ^*∗∗*^*p* < 0.001 at the two-tailed level, level of significance between the groups was analysed using separate least squares regression. ^#^95% CI was Bonferroni adjusted for the six subdomains of quality of life scores (i.e., 99.2%); ^##^Bonferroni adjusted statistical significance level for the six subdomains of quality of life scores (*α* = 0.008). Values are group mean ± SD.

## Data Availability

The group mean data are given in the paper. The individual data are available in the archives of the laboratory and can be obtained from the corresponding author on request.
